# Core components of infection prevention and control programs at the facility level in Georgia: key challenges and opportunities

**DOI:** 10.1186/s13756-020-00879-3

**Published:** 2021-02-24

**Authors:** Anna Deryabina, Meghan Lyman, Daiva Yee, Marika Gelieshvilli, Lia Sanodze, Lali Madzgarashvili, Jamine Weiss, Claire Kilpatrick, Miriam Rabkin, Beth Skaggs, Amy Kolwaite

**Affiliations:** 1 ICAP, Columbia University, Almaty, Kazakhstan; 2 International Infection Control Program, Division of Healthcare Quality Promotion, U.S. Centers for Disease Control and Prevention, Atlanta, USA; 3 South Caucasus Office, U.S. Centers for Disease Control and Prevention, Atlanta, USA; 4 MoLHSA/NCDC&PH, Atlanta, USA; 5 TEPHINET, Atlanta, GA USA; 6 Consultant to the World Health Organization, Geneva, Switzerland; 7 ICAP, Columbia University, New York, USA

**Keywords:** Infection prevention and control, WHO core components, Facility assessment, Georgia

## Abstract

**Background:**

The Georgia Ministry of Labor, Health, and Social Affairs is working to strengthen its Infection Prevention and Control (IPC) Program, but until recently has lacked an assessment of performance gaps and implementation challenges faced by hospital staff.

**Methods:**

In 2018, health care hospitals were assessed using a World Health Organization (WHO) adapted tool aimed at implementing the WHO’s IPC Core Components. The study included site assessments at 41 of Georgia’s 273 hospitals, followed by structured interviews with 109 hospital staff, validation observations of IPC practices, and follow up document reviews.

**Results:**

IPC programs for all hospitals were not comprehensive, with many lacking defined objectives, workplans, targets, and budget. All hospitals had at least one dedicated IPC staff member, 66% of hospitals had IPC staff with some formal IPC training; 78% of hospitals had IPC guidelines; and 55% had facility-specific standard operating procedures. None of the hospitals conducted structured monitoring of IPC compliance and only 44% of hospitals used IPC monitoring results to make unit/facility-specific IPC improvement plans. 54% of hospitals had clearly defined priority healthcare-associated infections (HAIs), standard case definitions and data collection methods in their HAI surveillance systems. 85% hospitals had access to a microbiology laboratory. All reported having posters or other tools to promote hand hygiene, 29% had them for injection safety. 68% of hospitals had functioning hand-hygiene stations available at all points of care. 88% had single patient isolation rooms; 15% also had rooms for cohorting patients. 71% reported having appropriate waste management system.

**Conclusions:**

Among the recommended WHO IPC core components, existing programs, infrastructure, IPC staffing, workload and supplies present within Georgian healthcare hospitals should allow for implementation of effective IPC. Development and dissemination of IPC Guidelines, implementation of an effective IPC training system and systematic monitoring of IPC practices will be an important first step towards implementing targeted IPC improvement plans in hospitals.

## Background

Inadequate infection prevention and control (IPC) practices in healthcare hospitals are a main driver of increasing rates of antimicrobial resistance (AMR) and healthcare-associated infections (HAIs) [5, 9, 15] and are a growing concern for health care and for public health worldwide [20]. Studies estimate that one in 18 hospitalized patients in Europe and one in 25 hospitalized patients in the United States has a HAI on any given day [17, 33].

While HAI data in Georgia are limited, studies suggest transmission of HAIs is an important problem [7, 14, 16]. Georgia has a high prevalence of hepatitis C virus (HCV) infection associated with healthcare exposures due to inadequate infection control [11, 23]. In response to the high HCV prevalence and growing concerns around AMR, the Georgia Ministry of Labor, Health, and Social Affairs (MoLHSA) instituted the National Strategy for Combating Antimicrobial Resistance and the Hepatitis C Elimination Program, which includes a focus on strengthening IPC and reducing healthcare transmission.

Healthcare transmission of AMR and HAIs can be prevented through comprehensive and robust IPC programs [4, 31, 32]. To support countries’ efforts to strengthen IPC, the World Health Organization (WHO) released their new evidence-based guidelines on IPC core components in 2017 [32, 36]. These guidelines cover eight areas of IPC and include 14 recommendations and best practice statements.

A number of MoLHSA decrees exist that describe facility-level IPC requirements and are treated as National IPC guidelines. However, the decrees are fragmented and only provide general IPC recommendations. Information about necessary infrastructure and implementation of IPC at Georgian healthcare hospitals is limited and only covers the built environment or HAI epidemiology and etiology. To address these gaps, ICAP at Columbia University in close collaboration with the U.S. Centers for Disease Control and Prevention (U.S. CDC), and WHO used a modified version of the draft WHO IPC Assessment Framework (IPCAF) tool to conduct a systematic assessment of IPC practices in Georgia. This study is the first formal and systematic assessment of IPC core components at the facility level in Georgia.

## Methods

### Study protocol

The study protocol was approved by the Institutional Review Boards (IRB) of Georgia’s National Center for Disease Control and Public Health (NCDC) and Columbia University Medical Center. This project was reviewed in accordance with the U.S. CDC human research protection procedures and was determined to be non-research. Participation in the study was voluntary and informed consent was sought from all participants, with the option to withdraw consent at any time. Respondents were informed that results of the assessment would be presented to the MoLHSA in the form of a summarized report with no data on individual hospitals included. There were no refusals to participate in the study. No compensation for participation was provided.

### Hospital selection

A random sample of inpatient hospitals, stratified by geographic region and service status (i.e. public, private), was selected to ensure proportional representation. Three multi-specialty hospitals from hospital networks not included in the random sample were added to ensure representation of all private hospital networks. Small hospitals, defined as multi-specialty hospitals with less than 20 beds and specialty hospitals with less than 10 beds, were excluded. Hospitals providing only psychiatric services or tuberculosis treatment were also excluded because of the specialized care and unique IPC issues.

### Pilot study and data collection

The assessment tool used was adapted from the IPCAF issued by WHO in 2018 to support the implementation of the WHO Guidelines on Core Components of IPC Programs at the National and Acute Health Care Facility Level [37, 38]. A study published in 2020 highlights that effective utilization of the IPCAF tool requires a deep understanding of the WHO terminology and underlying concepts to avoid misinterpretation and misreporting of data [34]. To improve quality of data and avoid biased reporting, the team adapted the IPCAF tool for the situational assessment in Georgia. For that, the study team conducted several meetings with local specialists involved in IPC and external IPC experts from the U.S. CDC and WHO to review the questions, select those that were relevant to Georgia, and add additional questions providing more details or verification. The revised questionnaires (Annex 2) were then transferred into ICAP’s online survey data collection system (e-Survey) and piloted at two hospitals located in the capital Tbilisi, not included in the study sample. Results of the pilot were used to revise the questionnaires and data collection procedures.

Data were collected during March 2018 by a team of local specialists involved in IPC implementation, monitoring, and training. All data collectors received a two-day training by ICAP at Columbia University in protocol implementation, interviewing techniques and ethical considerations. All healthcare facility assessments were conducted during a one-day visit by two study team members. The first part of the assessment consisted of key informant interviews, conducted in Georgian, with hospital managers and the facility IPC teams, and included a review of the facility’s available IPC-related documents. Individual and small group structured interviews were conducted at 41 hospitals and included 109 facility staff, including 51 facility managers and 58 IPC team members (i.e., nurses, epidemiologists, physicians). Disagreements around answers to the same questions for the same hospital were resolved by facilitating a discussion among hospital IPC team members to reconcile discrepancies until a final answer was agreed upon and recorded. During the second part of the assessment, the study team conducted a facility walk-through using observations to verify answers provided during the interviews. Data were entered into a tablet computer using e-Survey. Answers to open-ended questions were audio recorded and then transcribed in Georgian and translated into English for analysis.

### Data analysis and reporting

Descriptive analysis was conducted for categorical data using frequency analysis and cross-tabulation. Qualitative data from key informant interviews were grouped into meaningful patterns and/or themes through content and thematic analysis using NVivo^©^. Further analysis of each theme was undertaken using a three-step approach, “describe, compare, relate” [6]. Data from individual interviews were either linked with data from the document review and facility observations to allow for multidimensional descriptions of IPC core components at the facility level or integrated with each other to produce a fuller picture of IPC core components at the facility level [21].

A final written report was shared with the MoLHSA. A national IPC stakeholder meeting, which included national and facility leaders, was conducted by the MoLHSA in collaboration with the U.S. CDC and WHO. The meeting included a presentation of preliminary survey findings, expert opinion on interpretation of the data, and open discussions on the need to strengthen IPC and develop partnership between all levels to improve IPC implementation in Georgia.

## Results

The assessment included 41 hospitals (31 multi-specialty hospitals and 10 specialized hospitals), covering 15% of all hospitals in Georgia. Among these hospitals, the average bed capacity was 73 beds per facility (range 10–230 beds). Key assessment findings related to facility-level IPC system characteristics as recommended by WHO are discussed in the text below. Detailed assessment results are presented in Annex 1.

### IPC program components

Of the 41 hospitals participating in the assessment, 38 (93%) had an IPC program. However, none of the IPC programs had all the WHO-recommended elements including clearly defined objectives based on local epidemiology, annual IPC workplans, adequate improvement measures and targets, and a specified IPC budget.

All hospitals included in the assessment had an IPC team, 32 (78%) hospitals had more than one IPC team member and 34 (83%) hospitals had at least one full-time IPC specialist. At least one IPC team member in 27 hospitals (66%) had received some formal IPC training. During interviews, absence of dedicated, full-time IPC nurses, lack of IPC certified courses and limited professional development opportunities for IPC personnel were cited as key barriers to adhering to the WHO Core Component recommendations.

Of the hospitals included in the assessment, 39 (95%) hospitals reported having an IPC committee consisting of a multidisciplinary group that advises the IPC team. IPC committees at every facility included senior leadership (e.g., administrative director, the chief executive officer, medical director) and senior clinical staff (e.g., chief physician, chief of nursing). Additionally, IPC committees at 28 of the 39 hospitals (72%) included facility management staff, such as biosafety, water, sanitation, and hygiene (WASH) staff. Thirty-eight of the 39 hospitals (93%) reported their committee met at least once in the past 12 months. However, documentation of IPC committee meetings, as evidenced by meeting notes, was available at only in 19 of 38 (50%) hospitals.

Thirty-five (84%) of the 41 hospitals had access to microbiology laboratory within or outside of the facility for day-to-day use. Hospitals located in urban areas had more access to microbiology laboratories compared to rural hospitals, (77% vs. 23%).

### IPC training

Thirty-seven (90%) of 41 hospitals had conducted IPC trainings in the previous 12 months. Most hospitals trained clinical and non-clinical staff on IPC, however, ongoing IPC annual training for clinical staff was required at 54% of hospitals. Nine (22%) of 41 hospitals conducted IPC trainings for all clinical staff as part of new employee orientation in addition to mandatory refresher trainings at least annually. Eight (20%) of 41 hospitals conducted IPC trainings for all non-clinical staff during orientation as well as regular mandatory refresher trainings at least annually. During individual interviews, IPC focal persons mentioned lack of regular IPC training for clinicians and the need for technical assistance to develop IPC training programs at their facility as challenges to implementing IPC.

### IPC monitoring and audit

Seven (17%) hospitals had an IPC monitoring/audit plan available, however none of these plans had all the necessary elements, such as clear goals and objectives, tools to systematically collect data, clearly defined roles and responsibilities, and a work plan or schedule. Thirty-two of 41 (78%) hospitals reported conducting internal monitoring/audits in the last 3 months, 20 of 41 (49%) hospitals surveyed provided documentation of these monitoring/audits. Among these 20 hospitals, none conducted internal monitoring/audits at least once a month for each category of IPC practices. Only 17 (41%) conducted monitoring/audit in the past 12 months and shared the results with all cadres of facility staff, including clinical and non-clinical staff, IPC committee and facility management.

### HAI surveillance

Thirty-one of 41 (76%) hospitals reported conducting HAI surveillance. However, none of the hospitals reported having a system that include all HAI surveillance components recommended by WHO including a list of priority HAIs, standardized case definitions, standardized data collection and review methods, and clearly defined roles and responsibilities.

### IPC guidelines

Thirty-one (76%) of the 41 hospitals had IPC guidelines available, including 26 (63%) that used national guidelines, two (5%) that used international guidelines translated into Georgian, and three (7%) that used internal guidelines developed by their own facility staff. Of the total sample, 18 (44%) reported training their clinical staff on the IPC guidelines. Thirty-three (80%) hospitals had IPC SOPs available, 18 (55%) of which had facility-specific SOPs adapted by IPC personnel and clinical staff.

### Multimodal strategies

Facility use of multimodal strategies for hand hygiene (HH) and injection safety were assessed. Injection safety was specifically targeted given the high prevalence of HCV in Georgia. While all hospitals reported having reminders, posters, or other tools to promote hand hygiene, only 19 (46%) hospitals displayed them at all hand hygiene stations. Four (10%) hospitals used additional methods to improve team communication for hand hygiene across units. Twelve (29%) of hospitals reported having reminders, posters, or other tools to promote injection safety with only four hospitals (10%) had visible reminders, posters, or other tools to raise awareness of injection safety at all stations. Managers showed visible support and served as role models for hand hygiene in 23 (56%) hospitals and for injection safety in 21 (51%) hospitals.

### IPC infrastructure and supplies

Most hospitals reported having the basic infrastructure and supplies needed to conduct IPC including building features, such as energy and water supply (100%), bed occupancy limited to one patient per bed in all units (100%), adequate spacing (at least 1 m) ensured between beds in all units (88%), and functioning environmental ventilation available in all patient care areas (98%). Thirty-six hospitals (88%) had single rooms available for individual isolation. Thirty-seven hospitals (90%) had dedicated decontamination area and/or sterile supply department available and functioning, and 40 hospitals (98%) reported to have sterile and disinfected equipment ready for use every day and of sufficient quantity.

However, for many hospitals, responses to interview questions were inconsistent with infrastructure and supply observations. For example, 21(51%) hospitals reported having a daily record of cleaning, but the daily record was verified by data collectors’ observations in 13 (32%) hospitals. Similarly, 37 (90%) hospitals reported having functioning hand hygiene stations available at all points of care, but data collectors were only able to verify through their observations in 28 (68%) hospitals. Almost all hospitals (98%) reported having functional waste collection containers available at all waste generation points, however these were observed in only 29 (71%) hospitals.

In interviews, several facility managers from rural hospitals cited poor infrastructure (i.e., lack of rooms, need for complete renovation of premises, broken sewage systems) and absence of funding to improve infrastructure as leading factors preventing effective IPC implementation at their hospitals.

## Discussion

Overall, this study showed that the presence of an IPC program in Georgia does not directly correlate to a well-functioning facility-level IPC system where core IPC components are present.

Specifically, we found challenges in IPC-related staffing and training. While most hospitals had a sufficient number of designated IPC staff, nurses were part of the IPC team at only 78% of hospitals, despite recommendations for all IPC teams to include nurses [24]. In addition, IPC staff had received formal training at only 66% of hospitals surveyed; less than a third of hospitals routinely trained all new clinical and non-clinical staff as well as conducted mandatory IPC training at least annually. Only 76% of hospitals surveyed had any IPC guidelines, most were using national IPC guidelines that were not locally adapted, and only 58% of hospitals with IPC guidelines conducted related trainings. These findings correlate with research conducted previously at nine Georgian maternity hospitals showing that less than 70% of clinical staff received any type of training on HAI and that trainings conducted were limited to short seminars or ad hoc presentations [8]. Lack of effective capacity building for the healthcare workforce in Georgia is not unique to IPC,several other reviews have found inadequate training of healthcare providers to be a key challenge to quality health care services (Akhvlediani, Akhvlediani, & Kuchuloria, 2016; [16, 22, 29]. Suboptimal IPC education and training is also not unique to Georgia, as similar shortcomings were demonstrated during surveys conducted in high-income settings like Germany and Austria [1, 2].

Our findings also show that very few hospitals in Georgia used any systematic tools to routinely monitor IPC practices. Regular monitoring of IPC practices and timely feedback to all relevant staff is critical to prevent and control HAI at the facility-level [36]. Evidence shows a relationship between monitoring of hand-hygiene practices and reduced rates of HAI [10, 13, 25, 39], and WHO recommends monitoring all critical aspects of IPC, such as interventions to prevent catheter-related bloodstream infections and ventilator-associated pneumonia, as well as auditing of environmental cleaning procedures. Sharing of information with relevant facility staff was also rare. This is consistent with a 2016 review noting that due to limited training and monitoring, medical staff often neglected proper hand washing and use of personal protection equipment [3].

To our knowledge, this is the first systematic assessment of WHO’s IPC core components at Georgian health hospitals. Previously published studies focused on HAI and AMR epidemiology, including neonatal blood stream infections [16, 30], multi-drug resistant tuberculosis [19, 35], and specific pathogens such as antibiotic resistant *Staphylococcus aureus*, *Pseudomonas aeruginosa*, and *Enterococcus* spp. [26]. None of the published studies, however, used a systematic approach to evaluate the organization and implementation of IPC at the facility level.

There are limitations to this assessment. Hospitals were randomly selected to include those of different geographic locations, types, and sizes. Although the response rate for the selected facilities was 100%, specialized hospitals for treatment of psychiatric conditions and tuberculosis, specialized hospitals with less than 10 beds, and general hospitals with less than 20 beds were excluded from the study, therefore, the results cannot be considered nationally representative. While data collectors informed participants that results would not be used to evaluate individual hospitals and would not result in punitive actions, staff may have been reluctant to share deficiencies. For areas where direct observations were not made, the assessment team verified the answers by cross-checking the available hospital documentation, however, over-reporting of presence of certain IPC components still likely occurred. Despite efforts to provide contextual information to participants, confusion about new or unfamiliar concepts may have affected the accuracy of their answers.

Strengths of this assessment include its focus on the gap between IPC policy and IPC implementation, as well as its relatively large sample size and systematic approach to data collection. This survey highlights that the presence of an IPC committee or policy do not always translate into functioning IPC activities at hospitals in Georgia. The results also highlight the challenges that can occur even in a country with a national IPC strategy. They highlight the need for ongoing systems strengthening at both the facility level and the national level. Development and dissemination of IPC Guidelines, implementation of an effective IPC training system and systematic monitoring of IPC practices will contribute to improved IPC in the country.

## Conclusions

Our study shows that most of Georgian hospitals we surveyed have parts of an effective IPC program, namely an IPC committee, an IPC policy, designated staff and basic infrastructure in place; however, this does not translate into functional IPC activities. Georgia is reforming their healthcare system to ensure universal health coverage (UHC) and improve the quality of healthcare services, but achieving UHC with quality health services is not possible without an effective IPC system [31]. Endorsement of this study by the MoLHSA is an important step in Georgia's commitment to improving IPC and adhering to WHO’s IPC Core Components. There are resources available which provide practical tools for strengthening IPC programs at the national and facility level, based on the WHO IPC Core Components, and addressing the gaps identified during this assessment [37, 38].

Georgia is currently updating national IPC guidelines, based on international standards, but adapted to the Georgian context. This is an initial step to establishing national IPC standards, upon which to base the development of IPC trainings and an IPC monitoring system. A comprehensive IPC monitoring system at the facility and national level is critical to ensure compliance and guide future IPC improvements. Continuous improvement will require regular monitoring and use of IPC data.

## Data Availability

The authors are responsible for the data described in the manuscript and assure full availability of the study material upon request to the corresponding author.

## Annex 1: Assessment of IPC system characteristics

**Table:**

IPC facility characteristics (N=41)		n (%)
IPC program components		
Clearly defined responsibilities		21 (51)
Annual work plan		18 (44)
Clearly defined objectives		16 (39)
Measurable outcomes		2 (5)
Specifically allocated budget		4 (10)
Sufficient IPC budget		16 (39)
**IPC team components**		
IPC team includes more than one staff member		32 (78)
Members had received formal IPC training/education		27 (66)
Access to microbiology support		35 (84)
IPC Training		
**IPC training participants**		
Clinical staff		37 (90)
Non-clinical staff having contact with patients or wards		32 (78)
Family members, care-givers or visitors		1 (2)
**Frequency of IPC training**		
Staff trained as part of new employee orientation		
Clinical staff		14 (34)
Non-clinical staff		12 (29)
Ongoing mandatory training at least annually		
Clinical staff		22 (54)
Non-clinical staff		19 (46)
**Other characteristics of IPC training**		
IPC training included interactive sessions		22 (54)
Monitoring of which clinical and non-clinical staff had been trained in IPC		33 (80)
IPC Monitoring and audit		
IPC monitoring/audit plan		7 (17)
**Components of monitoring/audit plan**		
Clear goals and objectives		1 (2)
Tools to collect data in a systematic way		3 (7)
Clearly defined roles and responsibilities		0
Work plan or schedule		6 (15)
Monitoring results were used to make unit/facility-specific plans to improve IPC practices		18 (44)
**Frequency of internal monitoring/auditing**		
At least once within the past 3 months		32 (78)
At least once within the past 6 months		1 (2)
At least once within the past 12 months		0
More than 12 months ago or never		8 (20)
Conducted and documented internal monitoring/auditing within the past 12 months		20 (49)
**Hospitals conducting internal monitoring/audits at least once a month, by IPC practice**		
Hand Hygiene compliance		2 (5)
Consumption/usage of alcohol-based hand rub or soap		1 (2)
Injection safety		1 (2)
Waste management		1 (2)
Cleaning of ward environment		3 (7)
Disinfection and sterilization of medical equipment/instruments		2 (5)
Transmission-based precautions, isolation and cohorting (grouping) of patients		0
Consumption/usage of antimicrobial agents		1 (2)
Intravascular catheter insertion and/or care		1 (2)
Wound dressing change		0
**Sharing IPC monitoring/audit results (oral or written) with different staff types in the past 12 months**		
Clinical staff		19 (95)
Non-clinical staff that have direct contact with patients		17 (85)
Clinical managers/heads of department		19 (95)
IPC committee member		18 (90)
Non-clinical management (CEO, administration, board)		17 (85)
HAI surveillance		
Reported conducting HAI surveillance		31 (76)
**HAI surveillance components**		
List of priority HAIs which are major causes of morbidity and mortality in the facility		15 (37)
Standardized case-definitions (defined numerator and denominator)		13 (32)
Standardized data collection methods		14 (34)
Processes to review data quality		6 (15)
Clearly defined roles and responsibilities of staff involved in surveillance		6 (15)
Annual work plan and schedule		3 (7)
Surveillance system includes none of the above components		7 (22)
IPC guidelines		
**Types of IPC guidelines**		
International guidelines that have not been adapted to facility context (translated into Georgian)		2 (5)
National guidelines		26 (63)
Facility develops own guidelines		3 (7)
**IPC guideline training**		
Trainings included written information and/or oral instructions		18 (44)
Trainings included interactive sessions		14 (34)
Multimodal strategies		
**Hand hygiene**		
System change	Interventions to ensure the necessary infrastructure and continuous availability of supplies	33 (80)
	Interventions to ensure optimal use and accessibility and prevent human error	10 (24)
Education and training on IPC practices	Written information and/or oral instruction/e-learning	28 (68)
	Interactive training sessions	21 (51)
Monitoring of compliance and feedback	Audits of hand hygiene conducted	12 (29)
	Audit results shared and discussed with HCWs and key players	5 (12)
Communication and reminders	Reminders, posters, or other tools used to promote or raise awareness of hand hygiene	41 (100)
	Additional methods/initiatives to improve team communication across units/specialties	4 (10)
Safety climate and culture change	Managers/leaders show visible support and act as champions/role models	23 (56)
	Facility staff are empowered to participate in hand hygiene improvement activities	10 (24)
**Injection safety**		
System change	Interventions to ensure the necessary infrastructure and continuous availability of supplies	38 (93)
	Interventions to ensure optimal use and accessibility and prevent human error	9 (22)
Education and training on IPC practices	Written information and/or oral instruction/e-learning	31 (76)
	Interactive training sessions	14 (34)
Monitoring of compliance and feedback	Audits of injection safety conducted	5 (12)
	Audit results shared and discussed with HCWs and key players	2 (5)
Communication and reminders	Reminders, posters, or other tools used to promote or raise awareness of injection safety	12 (29)
	Additional methods/initiatives to improve team communication across units/specialties	0
Safety climate and culture change	Managers/leaders show visible support and act as champions/role models	21 (51)
	Facility staff are empowered to participate in injection safety improvement activities	10 (24)
IPC infrastructure and supplies		
Appropriate building features	Bed occupancy limited to one patient per bed in all units	41 (100)
	Patients never placed outside in corridor	41 (100)
	Adequate spacing (>1m) ensured between beds in all units	36 (88)
	Sufficient energy supply is available for all uses in all areas	41 (100)
	Functioning environmental ventilation available in all patient care areas	40 (98)
Water supply and sanitation	Water services available at all times and in sufficient quantities for all uses	39 (95)
	Reliable safe drinking water station present and accessible for staff, patients and visitors in all wards	40 (98)
	Functioning hand hygiene stations available at all points of care	37 (90)
	Sufficient number of toilets/latrines available	39 (95)
Personal protective equipment	Sufficient PPE* available per each cleaning/waste disposal staff	32 (78)
Decontamination of items, equipment and medical devices	Dedicated decontamination area and/or sterile supply department available and functioning	37 (90)
	Reliably have sterile and disinfected equipment ready for use every day and of sufficient quantity	40 (98)
Isolation capacity	Single rooms available for isolation	36 (88)
	Rooms available for cohorting patients with similar pathogens if single rooms are unavailable	6 (12)
Waste management structure and processes	Functional waste collection containers available for non-infectious, infectious, and sharps waste at all waste generation points	40 (98)
	Functional burial pit/fenced waste dump or municipal pick-up available for disposal of non-infectious waste	41 (100)
	Outsourced waste disposal, incinerator, or alternative treatment functional and available for infectious and sharps waste	41 (100)
	Wastewater safely managed using on-site treatment or sent to a functioning sewer system	39 (95)
Cleaning of the environment	Visible record of cleaning completed and signed daily	21 (51)
	Appropriate and well-maintained materials for cleaning available	38 (93)

## Annex 2A: Questionnaires used for semi-structured interviews with health managers

**Table:**

#	Question	Answer	Skip
1	Does your facility have an IPC programme?	1. No 2. Yes	
2	Does your facility have a budget specifically allocated to the IPC programme (e.g. to address IPC materials, administrative support, staff)?	1. No 2.Yes	
3	Is there an IPC team with one or more IPC staff with dedicated time for IPC activities?	No Yes	SKIP to 5
4	Does the IPC team have at least one full-time IPC specialist (a nurse or a doctor or an epidemiologist) working 100% on IPC?	1. All IPC staff work part-time on IPC 2. 1 or more full-time IPC specialist for 250 beds and less 3. Less than 1 full-time IPC specialist for 250 beds and less	
5	Do you have an established IPC Committee or an equivalent actively supporting the IPC team?	1. No 2. Yes	
6	When was the last time you led or participated in a meeting to discuss IPC-related objectives, targets and challenges?	1. Never 2. More than 3 months ago 3. Within the past 3 months	
7	Are there any vaccines that are required for healthcare workers at your facility? *Please mark all that apply*	1. No required vaccinations 2. Hepatitis B for all previously unvaccinated 3. Annual influenza vaccine 4. MMR for those with no serologic evidence of immunity or prior vaccinations 5. Other	
8	What do you consider the top challenges to implementing IPC at this facility?		
9	What would be your recommendations to improve IPC in this facility? Probe: At the national (maybe regulatory level)? Probe: At the facility level?		

## Annex 2B: Questionnaires used for semi-structured interviews with IPC FOCAL POINT/TEAM

**PART 1**

**Table:**

#	Question	Answer	Skip
1	Does your facility have an IPC programme? **Choose one answer** ***Please ask for all the documents that support the answer.*** ***Review the document(s) together with the respondents and answer questions 1A-C.***	1. No 2. Yes	
1A	Does the existing IPC programme have clearly defined responsibilities and annual work plan? **SELECT ALL THAT APPLY**	1. No 2. The existing program has clearly defined responsibilities 3. The existing program has annual work plan	
1B	Does the existing IPC programme have clearly defined IPC objectives for the facility? **Choose one answer**	1. No 2. Yes, IPC objectives, but there is no evidence that they are based on local epidemiology and priorities according to risk assessments 3. Yes, IPC objectives based on local epidemiology and priorities according to risk assessments	
1C	Does the existing IPC programme have clearly defined IPC measurable outcome indicators for the facility? **Choose one answer**	1. No 2. Yes, IPC measurable outcome indicators (or adequate measures for improvement) 3. Yes, IPC, measurable outcome indicators **and** future targets	
2	Does your facility have a budget specifically allocated to the IPC programme (e.g. to address IPC materials, administrative support, staff)? **Choose one answer**	1. No 2. Yes 3. Don’t know	
3	Do you consider the budget allocated for IPC as sufficient to cover your needs? **Choose one answer**	1. No 2. Yes	
4	Is there an IPC team with one or more IPC staff with dedicated time for IPC activities? **Choose one answer**	1. No 2. Yes	SKIP to 8
5	How many staff (nurses and/or doctors and/or epidemiologists and/or others) working on the IPC team? ***Please ask for a copy of the Terms of Reference for the Team or the IPC Focal point and the document that certifies their appointment.***	______epidemiologist ______nurses ______physicians ______others	
6	Does the IPC team have at least one full-time IPC specialist (a nurse and/or a doctor and/or an epidemiologist/other) working 100% on IPC? **Choose one answer** ***Please verify the answer based on the available documents.***	1. All IPC staff work part-time on IPC 2. 1 or more full-time IPC specialist for 250 beds and less 3. Less than 1 full-time IPC specialist for 250 beds and less	
7	Have members of your IPC team received formal IPC course training? **Choose one answer** ***Please ask for any documents that would verify the answer***	1. None were trained 2. Some were trained 3. All were trained	
8	What IPC professional development options for the IPC team are available at your facility? **SELECT ALL THAT APPLY**	1. No professional development offered 2. IPC members attend IPC-related conferences or workshops 3. IPC members attend IPC-related training courses	
9	Do you have an IPC Committee or an equivalent actively supporting the IPC team? **Choose one answer** ***Please ask for a copy of the document appointing members of an Infection Control Committee.***	1. No 2. Yes	SKIP to 12
10	Which, if any, of the following professional groups are represented or included in the IPC Committee or an equivalent? **SELECT ALL THAT APPLY** ***Please verify the answer based on the available documents.***	1. Facility management (e.g. administrative director, chief executive officer (CEO), medical director) 2. Senior clinical staff (e.g. chief physician, chief of nursing) 3. Other facility management [e.g. biosafety, waste, those tasked with addressing water, sanitation and health (WASH)]	
11	Did the committee meet in the past 12 months? **Choose one answer**	1. No 2. Yes	SKIP to 13
12	Did you keep notes for the IPC committee meetings conducted in the past 12 months? **Choose one answer** ***If yes, please ask for a copy of meeting notes from all the meetings conducted during the past 12 months and verify the answer.***	1. No 2. Yes, for some 3. Yes, for all	
13	When was the last time someone from the facility management, led or participated in a meeting to discuss IPC-related objectives, targets and challenges? **Choose one answer** ***Please verify the answer based on the available documents.***	1. Never 2. More than 3 months ago 3. Within the past 3 months	
14	Does your facility have microbiological laboratory support for routine day-to-day use? **Choose one answer**	1. No 2. No, but the facility has access to a clinical laboratory at another site 3. Yes, an on-site laboratory is available	
**IPC Trainings** **Next, I would like to discuss the IPC trainings available to your staff. We will cover trainings that are part of new employee orientation and continuous educational opportunities for existing staff, regardless of level and position, for example trainings for senior administration and housekeeping staff. Trainings can include classroom, e-learning, bedside, and simulation training. We will also discuss periodic evaluations conducted to determine the effectiveness of your facility’s training programmes and assess staff knowledge**			
15	Did this facility conduct any IPC trainings for clinical staff and others having contact with patients or wards during the past 12 months? **SELECT ALL THAT APPLY** ***Please ask for copies of all training materials (agenda and list of participants) from all IPC-related trainings conducted during the past 12 months***	1. No IPC trainings conducted at this facility 2. Yes, for health care workers (clinical staff) 3. Yes, for non-clinical staff with access to patients or wards at your facility (e.g. cleaners, auxiliary service staff, administrative and managerial staff) 4. Yes, for family members, other care-givers or visitors	SKIP to 23 SKIP to 23 if only 4 is marked
16	Does this facility keep track of which clinical and non-clinical staff have been trained in IPC? **Choose one answer** ***Please verify the answer based on the available documents. If there is no documented proof that they track training participation, please mark No***	1. No 2. Yes	
17	Did IPC trainings for clinical and non-clinical staff conducted during the past 12 months include interactive training sessions (simulations and/or bedside trainings)? **Choose one answer** ***Please verify the answer based on the available documents. If not verified, then mark a different answer***	1. No, interactive trainings only included written information and/or oral instructions and/or e-learning 2. Some trainings also included interactive sessions 3. All trainings included interactive sessions	
18	Who led IPC trainings for clinical and non-clinical staff conducted during the past 12 months? **SELECT ALL THAT APPLY** ***Please verify the answer based on the available documents.***	1. External trainers from outside the facility 2. IPC team members 3. Non-IPC personnel	
19	How did you assess the effectiveness of IPC trainings conducted during the past 12 months? **SELECT ALL THAT APPLY** ***Please verify the answer based on the available documents.***	1. No assessment 2. Pre/post test 3. Post-training survey for participants 4. Compliance monitoring of IPC practices	
20	In general, which statement best describes when IPC training for clinical staff at your facility is delivered? **SELECT ALL THAT APPLY** ***Please verify the answer based on the available documents (training registers, tracking sheets, prikazes, etc).***	1. All new HCWs are trained as part of new employee orientation 2. Ongoing regular training at least annually, but not mandatory 3. Ongoing mandatory training at least annually (not confirmed by any documentation) 4. Ongoing mandatory training at least annually (verified by documentation) 5. None of the above	
21	In general, which statement best describes when IPC training for non-clinical staff in your facility is delivered? **SELECT ALL THAT APPLY** ***Please verify this answer based on the available documents (training registers, tracking sheets, prikazes, etc).***	1. All new HCWs are trained as part of new employee orientation 2. Ongoing regular training at least annually, but not mandatory 3. Ongoing mandatory training at least annually (not confirmed by any documents) 4. Ongoing mandatory training at least annually (verified by documentation) 5. None of the above	
22	Do clinical trainings conducted at your facility for physicians working in specialty areas (for example, surgery or anesthesiology) include IPC? ***For example, if there is a line insertion training, would HH and skin prep standards be embedded in it, not just taught separately as IPC training?*** **Choose one answer** ***Please verify the answer based on the available documents.***	1. No clinical trainings for specialists conducted at the facility 2. Clinical trainings for specialists are conducted, but IPC is not included 3. Yes, in some trainings 4. Yes, in all trainings	
**IPC Monitoring and Audit** **One role of the IPC team is to monitor or audit IPC practices, and provide feedback to staff in order to improve the quality of care and practice. An example of this is conducting hand hygiene observations to monitor staff compliance with appropriate hand hygiene practices.**			
23	Does this facility have an internal IPC monitoring/audit plan with any of the following? **SELECT ALL THAT APPLY** ***Please verify the answer based on the available documents. Only select options that were verified by the document review.***	1. No facility monitoring/audit plan available 2. Yes with clear goals and objectives 3. Yes with tools to collect data in a systematic way (for example checklists) 4. Yes with clearly defined roles and responsibilities 5. Yes with work plan or schedule	
24	When was the last time there was an internal IPC monitoring/audit to assess compliance of any IPC practices at your facility? **Choose one answer**	1. Within the past 3 months 2. Within the past 6 months 3. Within the past 12 months 4. More than 12 months ago or never	SKIP to 27
24A	Did IPC staff document in any form implementation of monitoring/audits conducted within the past 12 months documented? ***Please ask for a copy of all the available IPC monitoring/audit reports conducted during the past 12 months***	1. No 2. Yes	SKIP to 27
25	During the past 12 months, how often did you conduct monitoring/auditing of different IPC practices listed below in questions 25A-J? **Choose one answer for each of the following questions 25A-J** ***Please verify the answers based on the available documents.***		
25A	Hand Hygiene Compliance (using any observation tools) ***If monitoring tools are not available (checklist and schedule), but facility staff claim to conduct monitoring frequently, mark*** *“****Periodically but no regular schedule”***	1. Not conducted 2. Periodically but no regular schedule 3. Weekly 4. Monthly 5. Every 6 months 6. Once a year	
25B	Consumption/usage of alcohol-based hand rub or soap ***If monitoring tools are not available (checklist and schedule), but facility staff claim to conduct monitoring frequently, mark*** *“****Periodically but no regular schedule”***	1. Not conducted 2. Periodically but no regular schedule 3. Weekly 4. Monthly 5. Every 6 months 6. Once a year	
25C	Injection safety ***If monitoring tools are not available (checklist and schedule), but facility staff claim to conduct monitoring frequently, mark*** *“****Periodically but no regular schedule”***	1. Not conducted 2. Periodically but no regular schedule 3. Weekly 4. Monthly 5. Every 6 months 6. Once a year	
25D	Waste management ***If monitoring tools are not available (checklist and schedule), but facility staff claim to conduct monitoring frequently, mark*** *“****Periodically but no regular schedule”***	1. Not conducted 2. Periodically but no regular schedule 3. Weekly 4. Monthly 5. Every 6 months 6. Once a year	
25E	Cleaning of the ward environment ***If monitoring tools are not available (checklist and schedule), but facility staff claim to conduct monitoring frequently, mark*** *“****Periodically but no regular schedule”***	1. Not conducted 2. Periodically but no regular schedule 3. Weekly 4. Monthly 5. Every 6 months 6. Once a year	
25F	Disinfection and sterilization of medical equipment/instruments ***If monitoring tools are not available (checklist and schedule), but facility staff claim to conduct monitoring frequently, mark*** *“****Periodically but no regular schedule”***	1. Not conducted 2. Periodically but no regular schedule 3. Weekly 4. Monthly 5. Every 6 months 6. Once a year	
25G	Transmission-based precautions, isolation and cohorting (grouping) of patients to prevent the spread of multidrug resistant organisms (MDRO) ***If monitoring tools are not available (checklist and schedule), but facility staff claim to conduct monitoring frequently, mark*** *“****Periodically but no regular schedule”***	1. Not conducted 2. Periodically but no regular schedule 3. Weekly 4. Monthly 5. Every 6 months 6. Once a year	
25H	Consumption/usage of antimicrobial agents ***If monitoring tools are not available (checklist and schedule), but facility staff claim to conduct monitoring frequently, mark*** *“****Periodically but no regular schedule”***	1. Not conducted 2. Periodically but no regular schedule 3. Weekly 4. Monthly 5. Every 6 months 6. Once a year	
25I	Intravascular catheter insertion and/or care ***If monitoring tools are not available (checklist and schedule), but facility staff claim to conduct monitoring frequently, mark*** *“****Periodically but no regular schedule”***	1. Not conducted 2. Periodically but no regular schedule 3. Weekly 4. Monthly 5. Every 6 months 6. Once a year	
25J	Wound dressing change ***If monitoring tools are not available (checklist and schedule), but facility staff claim to conduct monitoring frequently, mark*** *“****Periodically but no regular schedule”***	1. Not conducted 2. Periodically but no regular schedule 3. Weekly 4. Monthly 5. Every 6 months 6. Once a year	
26	During the past 12 months, how did you share results of these internal monitoring /auditing of IPC practices with the following facility staff listed in questions 26A-E? **SELECT ALL THAT APPLY**		
26A	Clinical staff?	1. Did not share 2. Shared orally during staff meetings 3. Shared in a form of a written report	
26B	Non-clinical staff that have direct contact with patients?	1. Did not share 2. Shared orally during staff meetings 3. Shared in a form of a written report	
26C	Clinical managers/heads of department?	1. Did not share 2. Shared orally during staff meetings 3. Shared in a form of a written report	
26D	IPC committee?	Did not share Shared orally during staff meetings Shared in a form of a written report	
26E	Non-clinical management (CEO, administration, board)?	1. Did not share 2. Shared orally during staff meetings 3. Shared in a form of a written report	
27	During the past 12 months, how often were your monitoring results used to make unit/facility- specific plans for the improvement of IPC practices? **Choose one answer** ***If yes, please ask to provide examples. Please verify the answers based on the examples provided.***	1. Always 2. Sometimes 3. Never	
28	Do you assess IPC safety cultural factors in your facility? **Show example of the surveys (Appendix 9)** **Choose one answer**	1. No 2. Yes	
**HAI Surveillance** **HAI surveillance programmes describe the incidence and prevalence of HAIs in your facility, detect outbreaks in particular wards or patient populations, guide IPC strategies and priorities, and assess the impact and effectiveness of interventions.**			
29	Does this facility conduct HAI surveillance? **Choose one answer** ***Please ask for all the available HAI surveillance guidelines or other documents. Review the documents and answer question 29A.***	1. No 2. Yes	SKIP to 38
29A	Does HAI surveillance include any of the following? ***Please verify the answers based on the available documents.*** **SELECT ALL THAT APPLY**	1. List of priority healthcare associated infections which are major causes of morbidity and mortality in the facility ***If prioritization process is not described in the document, please ask to describe the process used to identify infections which are major causes of morbidity and mortality in the facility*** 1. Standardized case-definitions (defined numerator and denominator) 2. Standardized data collection methods 3. Processes to review data quality (for example, assessment of case report forms, review of microbiology results, denominator determination, etc.) 4. Clearly defined roles and responsibilities of staff involved in surveillance 5. Annual work plan and schedule 6. None of the above	SKIP to 38
30	Are you conducting HAI surveillance in your facility for the following infection types listed in questions 30A-G? **Choose one answer** ***Please ask for all the available HAI surveillance reports.***		
30A	Infections or colonization caused by multidrug-resistant pathogens (non-susceptibility to at least one agent in three or more antimicrobial categories)?	1. No 2. Yes	
30B	Device-associated infections (for example, catheter-associated urinary tract infections, central line-associated bloodstream infections, peripheral-line associated bloodstream infections, ventilator-associated pneumonia)?	1. No 2. Yes	
30C	Surgical site infections?	1. No 2. Yes	
30D	Infections that may affect health care workers (for example, hepatitis B or C, HIV, influenza)?	1. No 2. Yes	
30E	Infections in targeted vulnerable patient populations (for example, neonates, intensive care unit, immunocompromised, burn patients)?	1. No 3. Yes	
30F	Local priority epidemic-prone infections (for example, norovirus, influenza, tuberculosis)?	1. No 2. Yes	
30G	Clinically-defined (based on symptoms) infections?	1. No 2. Yes	
31	What data sources do you use for your HAI surveillance? **SELECT ALL THAT APPLY** ***Please verify the answers based on the available documents.***	1. Discharge diagnosis data 2. Voluntary notification from physicians or nurses 3. Ward-based assessments (e.g., chart review, discussion with nurses or physicians, patient exam) 4. Laboratory-based assessment (e.g., review of blood cultures) 5. None of these types of surveillance	
32	Have staff conducting HAI surveillance been trained in basic epidemiology, surveillance and IPC (i.e. capacity to oversee surveillance methods and manage/analyze/interpret data)?	1. No 2. Yes	
33	During the past 12 months, how were your HAIs surveillance data shared with facility staff? **SELECT ALL THAT APPLY** ***Please ask for any available reports or staff meetings to verify the answers***	1. Not shared with facility staff 2. Written reports 3. Oral updates 4. Presentation	
34	How often, do you provide up-to-date HAIs surveillance information to the following groups listed in questions 34A-E? **Choose one answer for questions 34A-E** ***Please verify the answers based on the available documents***		
34A	Clinical staff?	1. Never 2. Quarterly 3. Half-yearly 4. Annually 5. Periodically but no regular schedule	
34B	Non-clinical staff that have direct contact with patients	1. Never 2. Quarterly 3. Half-yearly 4. Annually 5. Periodically but no regular schedule	
34C	Clinical managers/heads of department?	1. Never 2. Quarterly 3. Half-yearly 4. Annually 5. Periodically but no regular schedule	
34D	IPC committee?	1. Never 2. Quarterly 3. Half-yearly 4. Annually 5. Periodically but no regular schedule	
34E	Non-clinical management?	1. Never 2. Quarterly 3. Half-yearly 4. Annually 5. Periodically but no regular schedule	
35	Are HAIs surveillance data used to make unit/facility- specific plans for the improvement of IPC practices? **Choose one answer** ***Please ask to provide examples and verify the answer***	1. No 2. Yes	
36	What best describes the microbiology laboratory capacity available to support HAI surveillance in this facility? **Choose one answer**	1. Laboratory is able to differentiate between gram positive and gram negative strains but cannot identify the pathogen 2. The laboratory can identify pathogens (e.g. isolate identification) 3. The laboratory can identify pathogens and antimicrobial susceptibility patterns	
37	Do you use any informatics/IT tools to support your HAI surveillance (for example, electronic health records)?	1. No 2. Yes	
38	How often do you analyze and report antimicrobial drug resistance data? **Choose one answer**	1. Never or rarely 2. Regularly *(e.g. quarterly/ half a year /annually)*	
**IPC Guidelines** **Facility IPC guidelines provide recommendations for IPC practices in a facility and may be adapted from existing international and national standards. Guidelines are often broad and high-level while standard-operating-procedures are more detailed step-by-step instructions more specific to a certain setting**			
39	Does your facility have any IPC Guidelines? ***Please ask for all the IPC Guidelines available at the facility. Review the documents to verify the answer.***	1. No 2. Yes	SKIP to 44
40	Which statement best describes the process you use in this facility to develop or adapt IPC Guidelines? **Choose one answer**	1. Facility uses international guidelines that have not been adapted to facility context 2. Facility uses national guidelines 3. Facility develops its own guidelines	SKIP to 42 SKIP to 42
41	Who participates in the development and/or adaptation of the facility-level Guidelines? **SELECT ALL THAT APPLY**	1. IPC personnel 2. Senior facility leadership Clinical staff 3. Facility management (e.g. Biosafety, Waste, WASH (i.e those tasked with addressing water, sanitation and health) 4. Quality managers	
42	Do the facility training materials reflect the most updated IPC Guidelines? **Choose one answer**	1. No training materials available 2. No 3. Yes 4. Don’t know	
43	Please describe the process you use to train HCW on IPC guidelines when they are issued/updated? **Choose one answer** ***Please ask to see any training notes and/or list of participants and agenda from the last training.***	1. Trainings only included written information and/or oral instructions 2. Some trainings included interactive sessions 3. All trainings included interactive sessions	
44	Does your facility have any IPC SOPs? **Choose one answer** ***Please ask for a copy of all the SOPs available at the facility. Review the documents to verify the answer.***	1. No 2. Yes, SOPs not adapted to this facility 3. Yes, adapted to this facility	SKIP to 46 SKIP to 46
45	Who participates in the development and/or adaptation of the facility-level SOPs? **Choose one answer** **SELECT ALL THAT APPLY**	1. IPC personnel 2. Senior facility leadership 3. Clinical staff 4. Facility management (e.g. Biosafety, Waste, WASH (i.e those tasked with addressing water, sanitation and health) 5. Quality managers	
**Multimodal strategies** **The term multimodal strategy refers to the implementation of several elements or components in an integrated way with the aim of improving an outcome and changing behavior. This multimodal strategy includes components such as system change which is the availability of infrastructure and supplies to enable IPC practices; education and training of healthcare workers and other hospital staff; monitoring of infrastructure, practices, processes, outcomes, and providing data feedback; reminders in the workplace; and culture change within the facility. In other words, the strategy involves “building” the right system, “teaching” the right things, “checking” the right things, “selling” the right messages, and ultimately ”living” IPC throughout the entire health system.**			
46	For **hand hygiene** (HH) improvement activities, does your facility have any or all of the following elements listed in questions 46A-E? **SELECT ALL THAT APPLY for questions 46A-E**		
46A	**System change**	1. Element not included in work activities 2. Interventions to ensure the necessary infrastructure and continuous 3. Availability of supplies 4. Interventions to ensure optimal use and accessibility and prevent human error ***Please ask to provide examples to verify the answer***	
46B	**Education and training on hand hygiene practices**	1. Element not included in work activities 2. Written information and/or oral instruction and/or e-learning 3. Interactive training sessions (includes simulation and/or bedside training)	
46C	**Monitoring of HH compliance and feedback**	1. Element not included in work activities 2. Audits of hand hygiene conducted 3. Audit results shared and discussed with health care workers and key players	
46D	**Communications and reminders**	1. Element not included in work activities 2. Reminders, posters, or other tools used to promote or raise awareness of hand hygiene 3. Additional methods/initiatives to improve team communication across units and specialties (for example, multidisciplinary rounds?)	
46E	**Safety climate and culture change**	1. Element not included in work activities 2. Managers/leaders (i.e. head of the hospital, chief clinicians, head of nursing) show visible support and act as champions and role models, promoting an adaptive approach and strengthening a culture that supports hand hygiene 3. Facility staff (clinical and non-clinical) are empowered to participate in hand hygiene improvement activities	
47	How frequently is the WHO Hand Hygiene Self-Assessment Framework Survey conducted? **Choose one answer** ***Show the Survey to remind people what it is.*** ***Please verify the answers based on the available documents.***	1. Never 2. Periodically but not annually or on a regular schedule 3. At least annually	
48	As far as your **injection safety** improvement activities, does your facility have any or all of the following elements listed in questions 48A-E? **SELECT ALL THAT APPLY for questions 48A-E**		
48A	**System change**	1. Element not included in work activities 2. Interventions to ensure the necessary infrastructure and continuous availability of supplies 3. Interventions to ensure optimize use and accessibility and prevent human error ***Please ask to provide examples to verify the answer***	
48B	**Education and training on injection safety**	1. Element not included in work activities 2. Written information and/or oral instruction and/or e-learning only 3. Interactive training sessions (includes simulation and/or bedside training)	
48C	**Monitoring of injection safety compliance and feedback** **GIVE THE Injection Safety CHECKLIST as an example (Appendix 8)**	1. Element not included in work activities 2. Audits of injection safety conducted 3. Results shared and discussed with health care workers and key players	
48D	**Communications and reminders**	1. Element not included in work activities 2. Reminders, posters, or other tools to promote or raise awareness of injection safety 3. Additional methods/initiatives to improve team communication across units and disciplines (for example, by facilitating multidisciplinary rounds)	
48E	**Safety climate and culture change**	1. Element not included in work activities 2. Managers/leaders (head of the hospital, chief clinician and head of nursing) show visible support and act as champions and role models, promoting an adaptive approach and strengthening a culture that supports injection safety 3. Facility staff (clinical and non-clinical) are empowered to participate in injection safety improvement activities	
49	Are your quality improvement staff involved in IPC activities? **Choose one answer**	1. No quality improvement unit/staff at the facility 2. Quality improvement unit/staff available, but not involved in IPC 3. Quality improvement unit/staff available and involved in IPC	
**IPC Infrastructure, Staffing, Workload and Supplies** **Finally, I would like to ask a few questions about the facility’s infrastructure, and availability of staff and IPC supplies.**			
50	Are water services available at all times and of sufficient quantity for all uses (e.g., hand washing, drinking, personal hygiene, medical activities, sterilization, decontamination, cleaning and laundry)? **Choose one answer**	1. No, available on average < 5 days per week 2. Yes, available on average ≥ 5 days per week or every day but not of sufficient quantity 3. Yes, every day and of sufficient quantity	
51	Is a reliable safe drinking water station present and accessible for staff, patients and families at all times and in all locations/wards? **Choose one answer**	1. No, not available 2. Sometimes, or only available in some places or not available for all users 3. Yes, accessible at all times and for all wards/groups	
52	Is bed occupancy in your facility kept to one patient per bed? **Choose one answer**	1. No 2. Yes, but not in all departments 3. Yes, for all units including pediatrics/neonatal and emergency	
53	Do you place patients in beds outside of the room (in the corridor) **Choose one answer**	1. Never 2. Sometimes, or only in some departments	
54	Do you ensure adequate spacing of >1 meter between patient beds? **Choose one answer**	1. No 2. Yes, but not in all departments 3. Yes, for all units including pediatrics and emergency	
55	Do you have a responsible person/party to assess and respond when adequate bed capacity is exceeded? **Choose one answer**	1. No 2. Yes the clinical head of department is responsible 3. Yes, the hospital administration/management is responsible	
56	Are functioning hand hygiene stations (e.g., alcohol-based hand rub solution or soap and water with a basin/pan and clean single-use towels) available at all points of care? **Choose one answer**	1. No 2. Yes, stations present, but supplies are not always available 3. Yes, always available	
57	In your facility, are ≥ 4 toilets or improved latrines (clean and functional) available for outpatient settings or ≥ 1 per 20 users for inpatient settings? **Choose one answer**	1. Less than the required number of latrines available and functioning 2. Sufficient number present but not all functioning or insufficient 3. Sufficient number present and functioning (4 or more (outpatients) and one per 20 users (inpatients))	
58	In your health care facility, is sufficient energy/power supply available at day and night for all uses (for example, pumping and boiling water, sterilization and decontamination, incineration or alternative treatment technologies, electronic medical devices, general lighting of areas where health care procedures are performed to ensure safe provision of health care and lighting of toilet hospitals and showers)? **Choose one answer**	1. No, never available 2. Yes, sometimes or only in some of the mentioned areas 3. Yes, always and in all mentioned areas	
59	Is functioning environmental ventilation available in-patient care areas, including natural (using natural forces to vent air through windows/doors) or mechanical ventilation? **Choose one answer**	1. No 2. Yes, in some patient care services 3. Yes, in all patient care areas	
60	For floors and horizontal work surfaces, is there a visible record of cleaning, signed by the cleaners each day? **Choose one answer**	1. No record of floors and surfaces being cleaned 2. Record exists, but is not completed daily or is outdated 3. Yes, record completed daily	
61	Are appropriate and well-maintained materials for cleaning (for example, detergent, mops, buckets, etc.) available? **Choose one answer**	1. No materials available 2. Yes, available but not well maintained (not labeled, broken, or dirty, etc.) 3. Yes, available and well-maintained	
62	Do you have single patient rooms or rooms for cohorting (grouping based on common illness) patients with similar pathogens if the number of isolation rooms is insufficient or unavailable (for example, TB, measles, cholera)? **SELECT ALL THAT APPLY**	1. No single rooms and no rooms for cohorting (grouping) patients 2. Rooms suitable for patient cohorting available 3. Single rooms are available	
63	Do you have functional waste collection containers for non-infectious (general) waste, infectious waste and, sharps waste at all waste generation points? **Choose one answer**	1. No bins or separate sharps disposal 2. Separate bins present but lids missing or more than 3/4 full; or two bins (instead of three); or bins at some but not all waste generation points. 3. Yes, all three containers	
64	Is a functional burial pit/fenced waste dump or municipal pick-up available for disposal of non-infectious (non-hazardous/general waste)? **Choose one answer**	1. No pit or other disposal method used 2. Pit in facility but insufficient dimensions; pits/dumps overfilled or not fenced/locked; or irregular municipal waste pick up 3. Yes	
65	Is outsourced waste disposal or an incinerator or alternative treatment for infectious and sharp waste (for example, an autoclave) functional and of a sufficient capacity? **Choose one answer**	1. No, none present 2. Yes	
66	Are at least two pairs of household cleaning gloves and one pair of overalls or apron and boots in a good state and available for each cleaning and waste disposal staff member? **Choose one answer**	1. No, not available 2. Yes, available but in poor condition 3. Yes, in good condition	
67	Is wastewater safely managed using on-site treatment (for example, septic tank followed by drainage pit) or sent to a functioning sewer system? **Choose one answer**	1. No, not present 2. Yes, available but in poor condition 3. Yes, in good condition	
68	Does your health care facility provide a dedicated decontamination area and/or sterile supply department for the decontamination and sterilization of medical devices and other items/equipment? **Choose one answer**	1. No, not present 2. Yes, present, but not functioning 3. Yes	
69	Do you reliably have sterile and disinfected equipment ready for use? **Choose one answer**	1. Available on average < 5 days per week 2. Available on average ≥ 5 days per week or every day, but not of sufficient quantity 3. Available every day and of sufficient quantity	
70	Are disposable items available when necessary? (for example, injection safety devices (such as sharps injury protection syringes and reuse prevention syringes), examination gloves) **Choose one answer**	1. Not available 2*. Only* sometimes available 3. Continuously available

**Table:**

**General questions** **Before we go around the facility, we would like to ask you a couple of more general questions.**	
71	What do you consider the top challenges to implementing IPC at this facility?
72	What would be your recommendations to improve IPC in this facility?

**PART 2**

The checklist should be completed by the assessment team based on direct observations during a random walk around the facility. It is recommended that the random walk will include visits to one ICU (if available) and a typical ward (department). In case of hospitals that have pediatric and adult wards, the team needs to visit one adult ward and one pediatric ward. In each ward, the team must check a minor procedures room and 2–5 patient rooms.

Please check the availability of the following:

**Table:**

73	Visible reminders, posters, or other tools to promote or raise awareness of hand hygiene **Choose one answer**	1. Not available 2. Available at some hand hygiene stations observed 3. Available at all hand hygiene stations observed
74	Visible reminders, posters, or other tools to promote or raise awareness of injection safety **Choose one answer**	1. Not available 2. Available at some units/wards/departments observed 3. Available at all units/wards/departments observed
75	Any patients in beds outside of the room (in the corridor)	1. No 2. Yes
76	More than 1 meter between patient beds?	1. No 2. Yes, but not in all departments 3. Yes, for all units including pediatrics and emergency
77	Hand hygiene stations (e.g., alcohol-based handrub solution or soap and water with a basin/pan and clean single-use towels) available at all points of care? **Choose one answer**	1. Not available 2. Yes, stations present, but supplies are not always available 3. Yes, always available
78	Functional waste collection containers for non-infectious (general) waste, infectious waste and, sharps waste at all waste generation points?	1. No bins or separate sharps disposal 2. Separate bins present but lids missing or more than 3/4 full; or two bins (instead of three); or bins at some but not all waste generation points. 3. Yes, all three containers
79	For floors and horizontal work surfaces, a visible record of cleaning, signed by the cleaners each day	1. No record of floors and surfaces being cleaned 2. Record exists, but is not completed daily or is outdated 3. Yes, record completed daily

## Abbreviations

AMR: Antimicrobial resistance
DQA: Data quality audit
HAI: Health care-associated infections
IICP: International Infection Control Program
IPCAF: IPC Situational analysis Framework
IPC: Infection prevention and control
IRB: Institutional review board
MoLHSA: Ministry of Labor Health and Social Affairs of Georgia
NCDC & PH: National Centers for Disease Control & Public Health, Georgia
PI: Principal investigator
SOP: Standard operating procedure
U.S. CDC: U.S. Centers for Disease Control and Prevention
WHO: World Health Organization
